# Targeting WISP1 to sensitize esophageal squamous cell carcinoma to irradiation

**DOI:** 10.18632/oncotarget.3358

**Published:** 2015-01-31

**Authors:** Hongfang Zhang, Honglei Luo, Zhaoyang Hu, Jin Peng, Zhenzhen Jiang, Tao Song, Bo Wu, Jing Yue, Rongjing Zhou, Ruifei Xie, Tian Chen, Shixiu Wu

**Affiliations:** ^1^ Hangzhou Cancer Institution, Hangzhou Cancer Hospital, Hangzhou, China; ^2^ Department of Radiotherapy, Huai'an First People's Hospital, Huai'an, China; ^3^ Department of Pathology, Hangzhou Cancer Hospital, Hangzhou, China; ^4^ Department of Bio-Informatics, Hangzhou Cancer Hospital, Hangzhou, China

**Keywords:** ESCC, radioresistance, WISP1, prognostic significance, mechanisms

## Abstract

Radiotherapy is a primary treatment modality for esophageal squamous cell carcinoma (ESCC). However, most of patients benefited little from radiotherapy due to refractory radioresistance. We found that WISP1, a downstream target gene of Wnt/β-catenin pathway, was re-expressed in 67.3 % of ESCC patients as an oncofetal gene. Expression of WISP1 predicted prognosis of ESCC patients treated with radiotherapy. Overall survival in WISP1-positive patients was significantly poorer than in WISP1-negative patients. Serum concentration of WISP1 after radiotherapy reversely correlated with relapse-free survival. Gain and loss of function studies confirmed that WISP1 mediated radioresistance both in esophageal squamous cancer cells and in xenograft tumor models. Further studies revealed that WISP1 contributed to radioresistance primarily by repressing irradiation-induced DNA damage and activating PI3K kinase. LncRNA BOKAS was up-regulated following radiation and promoted WISP1 expression and resultant radioresistance. Furthermore, WISP1 facilitated its own expression in response to radiation, creating a positive feedback loop and increased radioresistance. Our study revealed WISP1 as a potential target to overcome radioresistance in ESCC.

## INTRODUCTION

Esophageal carcinoma is the sixth leading cause of cancer-related death and eighth in incidence worldwide [1]. Esophageal squamous cell carcinoma (ESCC), one main pathological subtype of esophageal carcinoma, prevails in Asia and South America. Radiotherapy is recommended as a primary treatment modality for ESCC. However, the prognosis is dismal with less than 20 % of 5-year survival because of tumor recurrence or metastasis which is primarily mediated by residual therapy-resistant cells [2, 3]. Tumor radioresistance is very complex and heterogeneous. Several lines of evidences have suggested that gene mutation or aberrant activation of critical signaling pathways within irradiated tumor cells contributed to radioresistance by enhancement of DNA damage repair response [4-6]. Wnt/β-catenin pathway plays driving roles in cell growth, differentiation and survival. When aberrantly activated, Wnt/β-catenin pathway was demonstrated to mediate radioresistance in glioblastoma, breast cancer and head and neck cancer [7-9]. However, direct blockage of Wnt/β-catenin pathway to overcome tumor radioresistance may cause some side effects on normal physiological processes outside the tumor. A mouse experiment has shown that a single injection of Wnt signaling inhibitor DKK1 led to proliferation inhibition of small intestine and colon, and subsequent architectural degeneration and death of most of mice [9]. Thus, special inhibition of Wnt signaling pathway may better control tumor progression and simultaneously exert less side effect on normal tissues.

WISP1 was identified as a Wnt1 and β-catenin responsive gene in 1998 through subtractive hybridization of mouse mammary epithelial cell line C57MG and Wnt-1-expressing C57MG cells [10]. It was mainly expressed during embryonic development and was seldom expressed in adult normal tissues. However, WISP1 was reported to be re-expressed under devastating diseased conditions such as cancer or fibrosis [11]. Forced expression of WISP1 in normal rat kidney fibroblast cells led to accelerated cell proliferation, morphological transformation and *in vivo* tumorigenesis [12]. Furthermore, WISP1 was demonstrated to inhibit programmed cell death by up-regulation of Bcl-xl expression and inhibition of cytochrome c release [13]. In ESCC, WISP1 was discovered to be highly expressed in cancer tissues compared with in adjacent benign tissues, and its expression had an inverse correlation with the prognosis of patients [14]. However, the exact roles of WISP1 in ESCC progression were poorly elucidated. In our study, we found WISP1-positive ESCC patients had significantly poorer prognosis than those WISP1-negative patients after radiotherapy. Furthermore, serum concentration of WISP1 after radiotherapy was significantly reversely associated with relapse-free survival. Gain and loss of function studies confirmed that WISP1 mediated radioresistance both in ESCC cells and in xenograft tumor models. Furthermore, WISP1 was discovered to mediate radioresistance primarily by repression of irradiation-induced DNA damage and activation of PI3K kinase. The positive feedback loop of WISP1 expression in response to radiation also enhanced radioresistance. In conclusion, our data highlighted WISP1 as a highly attractive target to radiosensitize ESCC.

## RESULTS

### WISP1 as an oncofetal gene predicted poor prognosis of ESCC patients after surgery

**Fig.1:**
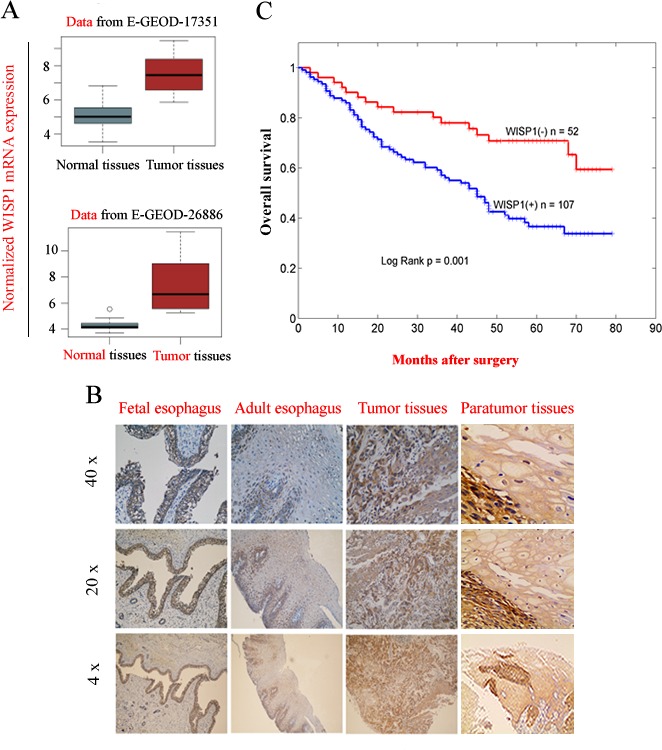
WISP1 as an oncofetal gene was a marker of poor prognosis of ESCC patients after surgery A. WISP1, a downstream target gene of Wnt/β-catenin pathway was highly expressed at mRNA level in ESCC tissues compared with in matched normal tissues by bio-informatics analysis of the data from GSE17351 and GSE26886. B. The expression of WISP1 protein in 22-weeks fetal esophagus, normal adult esophagus, ESCC tissues and matched non-neoplastic tissues by IHC analysis. C. Kaplan–Meier analysis showed that overall survival of WISP1-positive ESCC patients was significantly poorer than those WISP1-negative patients after surgery. The grading of WISP1 expression was described in “*Materials and Methods*”.

By bio-informatics analysis of GEO datasets in PUBMED database, Wnt/β-catenin pathway that controls cell fate via multiple mechanisms was found to be constitutively activated in esophageal carcinoma tissues compared with in adjacent normal tissues (Supplementary Fig. S1). Furthermore, we found WISP1, a downstream target gene of Wnt/β-catenin pathway, was significantly highly expressed in ESCC tissues compared with in adjacent normal tissues (*P*=0.037 in GSE17351; *P*=0.029 in GSE26886) (Fig. 1A). To uncover the exact expression pattern of WISP1 in ESCC, we compared its level of protein expression in 22-weeks fetal esophagus, normal adult esophagus, ESCC tissues and matched non-neoplastic tissues by immunohistochemical analysis (IHC). We found WISP1 was highly expressed in fetal esophagus and silenced in normal adult esophagus, but it was re-expressed in 159 primary ESCC patients with 67.3 % of positive ratio compared with 7.6 % of positive ratio in matched non-neoplastic tissues (*P*=0.0042), suggesting that WISP1 is an oncofetal gene in ESCC (Fig. 1B and Supplementary Fig. S2A). Furthermore, IHC analysis of tissue chips consisting of other 50 ESCC specimens also confirmed that WISP1-positive ratio in cancer tissues was significantly higher than in matched non-neoplastic tissues (65.8 % *vs* 13.4 %, *P*=0.004) (Supplementary Fig. S2B and S2C). Survival analyses showed that WISP1-positve ESCC patients (n=107) had significantly poorer prognosis than those WISP1-negative patients (n=52) after surgery (Fig. 1C). This finding was also supported by the study by Hideo Baba *et al* where WISP1 was discovered as a marker of poor prognosis of ESCC patients after surgery [14].

### WISP1 predicted poor prognosis of ESCC patients treated with radiotherapy

**Fig.2:**
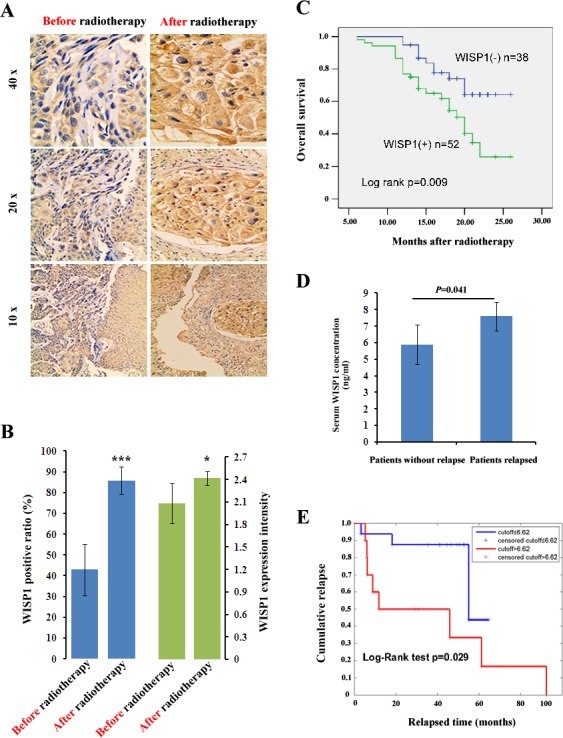
WISP1 was a prognostic factor of ESCC patients treated with radiotherapy A. A representative result of WISP1 expression in ESCC tissues before radiotherapy and after 60 Gy of radiation in 30 fractions by IHC analysis. B. Quantitative analysis of the intensity and positive ratio of WISP1 expression in ESCC tissues (n=12) before radiotherapy and after 60 Gy of radiation in 30 fractions. The grading of WISP1 expression was described in “*Materials and Methods*”. ***P<0.005, *P<0.05, compared with before radiotherapy. C. Kaplan–Meier analysis showed that overall survival of WISP1-positive patients (n=52) after radiotherapy was significantly poorer than those WISP1-negative patients (n=38). D. ELISA assay showed that serum concentration of WISP1 after radiotherapy was significantly higher in relapsed ESCC patients (n=12) than in those patients without relapse (n=15). E. Serum concentration of WISP1 after radiotherapy was significantly associated with relapse-free survival of ESCC patients.

Since WISP1 was defined as an oncofetal gene in ESCC, we investigated whether it was involved in tumor radioresponse. By IHC analysis of 12 cancer biopsy specimens, the intensity of WISP1 expression after 60 Gy of radiation in 30 fractions was found to increase to score of 2.4167 from score of 2.0833 before radiotherapy (*p*=0.0394) (Fig. 2A and 2B). Furthermore, the WISP1-positive ratio also increased to 85.83 % after 60 Gy of radiation in 30 fractions in comparison with 42.92 % before radiotherapy (*P*=0.0026) (Fig. 2A and 2B). These results indicated that WISP1 expression was significantly up-regulated after multiple fractionated radiation, and those WISP1-positive cells may be more radioresistant than those WISP1-negative cells. To confirm our hypothesis, we investigated whether WISP1 expression was associated with overall survival of ESCC patients treated with radiotherapy. Survival analysis showed that WISP1-positive patients after radiotherapy (n=52) had significantly poorer prognosis than those WISP1-negative patients (n=38) (Fig. 2C). By ELISA assay, serum concentration of WISP1 was also detected in 27 ESCC patients before radiotherapy and after 40 Gy of radiation in 20 fractions. Although serum concentration of WISP1 before radiotherapy showed no significant difference between patients with or without tumor relapse (data not shown), significant elevation of WISP1 concentration was detected in relapsed patients (n=12) than in patients without relapse (n=15) (*P*=0.041) (Fig. 2D). Furthermore, serum WISP1 concentration after radiotherapy was found to significantly correlate with relapse-free survival of ESCC patients (p=0.029) (Fig. 2E and Supplementary Fig. S2D). These results revealed WISP1 as a marker of poor prognosis of ESCC patients treated with radiotherapy. The clinicopathological characteristics of each cohort of patients analyzed above were summarized in Supplemental Information.

### WISP1 mediated radioresistance in ESCC cells

**Fig.3:**
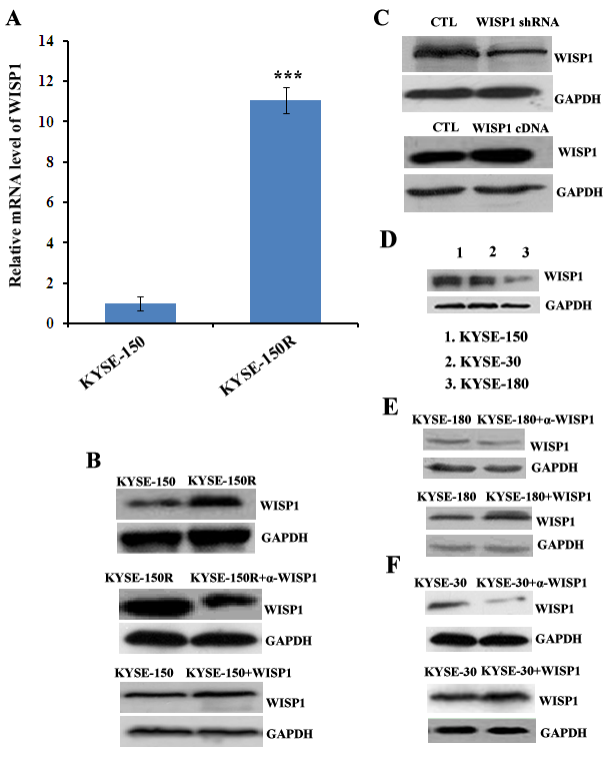
The expression of WISP1 in multiple ESCC cells A. The relative mRNA level of WISP1 in radioresistant cell KYSE-150R and in its parental cell KYSE-150 by qRT-PCR analysis. ***P<0.005, compared with KYSE-150 cell. B. Western blotting analysis of WISP1 expression in KYSE-150 cell treated with or without 2 μg/mL of recombinant WISP1 protein for 24 h and in KYSE-150R cell treated with or without 4 μg/mL of anti-WISP1 antibody α-WISP1 for 24 h. C. Western blotting analysis of WISP1 expression in KYSE-150 cell transduced with lentivirus vector carrying WISP1-targeting shRNA or WISP1 cDNA. D. Western blotting analysis of WISP1 expression in ESCC cells KYSE-150, KYSE-30 and KYSE-180. E. Western blotting analysis of WISP1 expression in KYSE-180 cell treated with or without 4 μg/mL of anti-WISP1 antibody α-WISP1 or 2 μg/mL of recombinant WISP1 protein for 24 h. F. Western blotting analysis of WISP1 expression in KYSE-30 cell treated with or without 4 μg/mL of anti-WISP1 antibody α-WISP1 or 2 μg/mL of recombinant WISP1 protein for 24 h. GAPDH served as a loading control in all of the western blotting analysis above.

**Fig.4:**
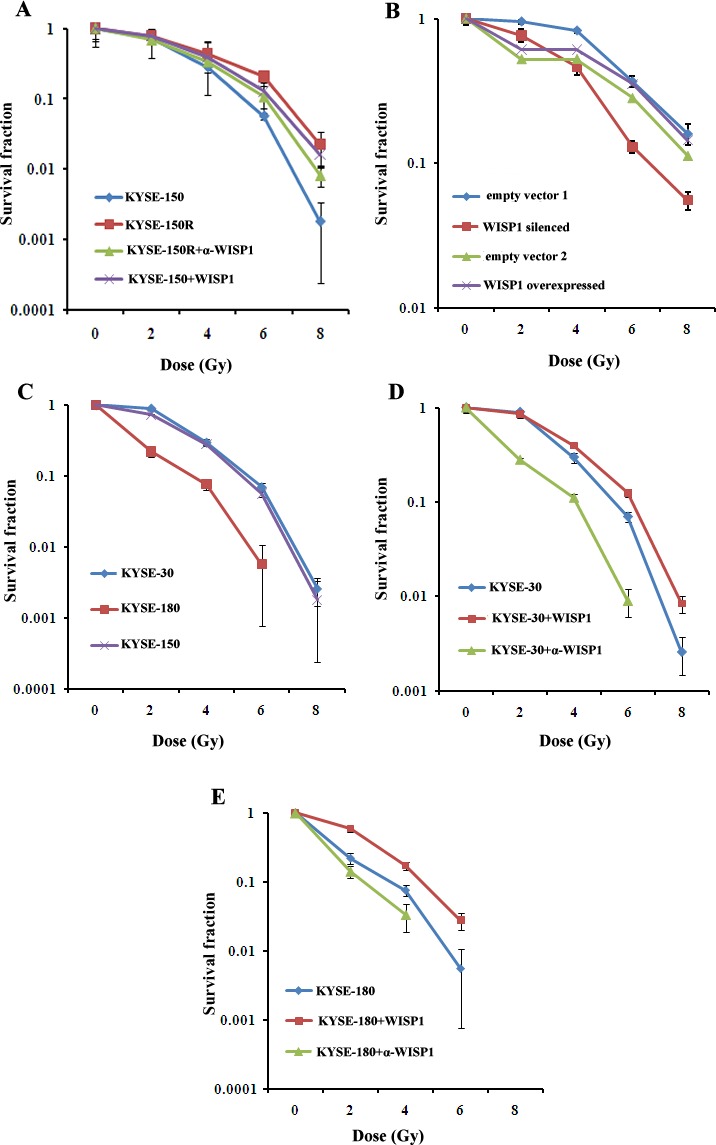
WISP1 mediated radioresistance in ESCC cells A. The radiosensitivity of KYSE-150R cell with or without treatment with 4 μg/mL of anti-WISP1 antibody α-WISP1 24 h before radiation, and the radiosensitivity of KYSE-150 cell with or without treatment with 2 μg/mL of recombinant WISP1 protein 24 h before radiation. B. The radiosensitivity of KYSE-150 cell with WISP1 silenced or overexpressed by stable transduction of WISP1-targeting shRNA or WISP1 cDNA. Empty vector 1 referred to empty lentivirus vector without carrying WISP1-targeting shRNA. Empty vector 2 referred to empty lentivirus vector without carrying WISP1 cDNA. C. The radiosensitivity of KYSE-30, KYSE-180 and KYSE-150 cells. D. The radiosensitivity of KYSE-30 cell treated with or without 4 μg/mL of anti-WISP1 antibody α-WISP1 or 2 μg/mL of recombinant WISP1 protein 24 h before radiation. E. The radiosensitivity of KYSE-180 cell with or without treatment with 4 μg/mL of anti-WISP1 antibody α-WISP1 or 2 μg/mL of recombinant WISP1 protein 24 h before radiation. The radiosensitivity of the above cells was detected at 0 Gy, 2 Gy, 4 Gy, 6 Gy and 8 Gy of radiation by clonogenic survival assay.

In our previous study, we had successfully established a radioresistant esophageal squamous cancer cell line KYSE-150R from its parental cell line KYSE-150 by multiple fractionated radiation [15]. In view of WISP1 as a marker of poor prognosis of ESCC patients treated with radiotherapy, we investigated whether its expression was up-regulated in radioresistant KYSE-150R cells. We found WISP1 expression in KYSE-150R cells was significantly up-regulated at both mRNA and protein levels compared with in KYSE-150 cells (Fig. 3A and Fig. 3B). When down-regulating WISP1 expression by treatment with 4 μg/mL of WISP1-specific antibody 24 h before radiation, the radioresistance of KYSE-150R cells was significantly reversed, suggesting that WISP1 may exert functional contribution to the radioresistance (Fig. 3B and Fig. 4A). Up-regulation of WISP1 expression by exposure to 2 μg/mL of recombinant WISP1 protein 24 h before radiation or stable transduction of WISP1 cDNA conferred KYSE-150 cells significant radioresistance (Fig. 3B and 3C; Fig. 4A and 4B). Furthermore, the radiosensitivity of KYSE-150 cells significantly increased when down-regulating WISP1 expression by stable transduction of WISP1-targeting shRNA (Fig. 3C and 4B). These data revealed that WISP1 was a critical mediator of radioresistance in KYSE-150R cells and KYSE-150 cells. To further confirm the causal relationship of WISP1 and radioresistance, two other esophageal squamous cancer cell lines KYSE-180 and KYSE-30 between which the level of endogenous WISP1 expression showed great difference have been employed (Fig. 3D). We found KYSE-180 cells with the lowest level of WISP1 expression were most radiosensitive in comparison with KYSE-150 cells and KYSE-30 cells (Fig. 4C). There was no significant difference in the radiosensitivity of KYSE-150 cells and KYSE-30 cells between which the level of WISP1 expression was approximately the same (Fig. 4C). Furthermore, we found down-regulation of WISP1 expression by treatment with 4 μg/mL of WISP1-specific antibody 24 h before radiation significantly radiosensitized both KYSE-30 cells and KYSE-180 cells (Fig. 3E and 3F; Fig. 4D and 4E). On the contrary, up-regulation of WISP1 expression by treatment with 2 μg/mL of recombinant WISP1 protein 24 h before radiation induced both KYSE-30 cells and KYSE-180 cells to display significant radioresistance (Fig. 3E and 3F; Fig. 4D and 4E). These above results together proved that WISP1 was a key mediator of radioresistance in ESCC cells.

### WISP1 mediated radioresistance in xenograft tumor models

**Fig.5:**
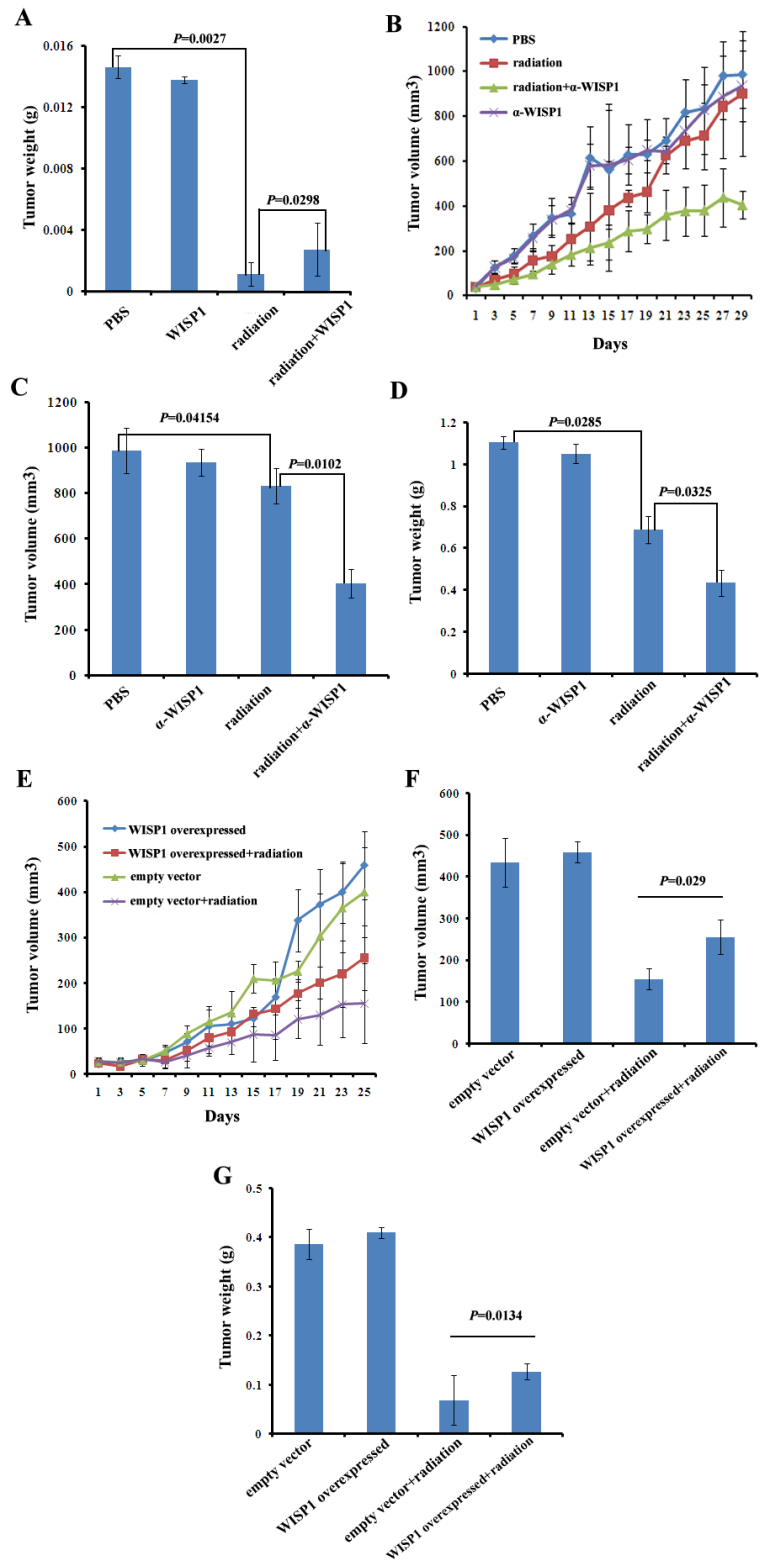
WISP1 mediated radioresistance in xenograft tumor models A. The weight of KYSE-180 xenograft tumors after treatment with PBS, fractionated radiation alone (12 Gy in three fractions every four days), recombinant WISP1 protein alone (2 μg/mL daily for 12 consecutive days) or fractionated radiation combined with recombinant WISP1 protein (fractionated radiation was performed at day 4, 8 and 12 after injection of 2 μg/mL of WISP1 protein had began at day 1 for 12 consecutive days). The weight of tumors was measured 10 days after the end of treatment. B. The growth curves of KYSE-30 xenograft tumors receiving different treatments including PBS, fractionated radiation alone (12 Gy in three fractions every four days), anti-WISP1 antibody α-WISP1 (4 μg/mL daily for 12 consecutive days) alone and fractionated radiation combined with α-WISP1 (fractionated radiation was performed at day 4, 8 and 12 after injection of 4 μg/mL of α-WISP1 had began at day 1 for 12 consecutive days). Tumor volume was calculated as described in “*Materials and Methods*”. C. The volume of xenograft tumors in “B” on day 29. D. The weight of xenograft tumors in “B” on day 29. E. The growth curve of KYSE-150 xenograft tumors transduced with empty vector or WISP1 cDNA after treatment with or without 12 Gy of radiation in three fractions every four days. Tumor volume was calculated as described in “Materials and Methods”. F. The volume of tumors in “E” on day 25. G. The weight of tumors in “E” on day 25.

To further confirm the causal relationship of WISP1 and radioresistance *in vivo*, KYSE-180 xenograft tumors with low level of WISP1 expression had been established in BALB/c nude mice. The tumors were treated with fractionated radiation alone or combined with recombinant WISP1 protein. PBS-treated tumors were used as a control. We found fractionated radiation alone (12 Gy in three fractions) significantly inhibited xenograft tumor weight in comparison with PBS treatment (0.00115 g *vs* 0.01465 g, *P*=0.0027) (Fig. 5A). However, the combination treatment with fractionated radiation and recombinant WISP1 protein (fractionated radiation was performed at day 4, 8 and 12 after injection of 2 μg/mL of WISP1 protein had began at day 1 for 12 consecutive days) induced significantly increased tumor weight compared with fractionated radiation alone (0.00275 g *vs* 0.00115 g, *P*=0.0298). Treatment with WISP1 alone did not have significant promotion effect on tumor weight in comparison with PBS treatment (0.01465 g *vs* 0.0138 g, *P*=0.0629) (Fig. 5A). These results indicated that up-regulation of WISP1 expression in KYSE-180 xenograft tumors significantly enhanced radioresistance.

KYSE-30 xenograft tumors with high level of WISP1 expression had also been developed in BALB/c mice and exposed to indicated treatments. We found fractionated radiation alone (12 Gy in three fractions) significantly inhibited tumor volume and weight compared with PBS treatment (830.8727 mm^3^ *vs* 987.9588 mm^3^, *P*=0.04154; 0.6882 g *vs* 1.1038 g, *P*=0.0285) (Fig. 5B, 5C and 5D). Moreover, the tumor growth inhibitory effect of fractionated radiation was further significantly enhanced when combined with anti-WISP1 antibody (fractionated radiation was performed at day 4, 8 and 12 after injection of 4 μg/mL of anti-WISP1 antibody had began at day 1 for 12 consecutive days) (409.9544 mm^3^*vs* 830.8727 mm^3^, *P*=0.0102; 0.4348 g *vs* 0.6882 g, *P*=0.0325). Treatment with anti-WISP1 antibody alone had no significant inhibitory effect on tumor growth (934.9269 mm^3^ *vs* 987.9588 mm^3^, *P*=0.1153; 1.0508 g *vs* 1.1038 g, *P*=0.3153) (Fig. 5B, 5C and 5D). These results indicated that down-regulation of WISP1 expression could significantly radiosensitize KYSE-30 xenograft tumors.

Furthermore, we also investigated whether up-regulation of WISP1 expression by stable transduction of WISP1 cDNA in lentivirus vector could reduce the radiosensitivity of KYSE-150 xenograft tumors. The tumors transduced with empty vector were used as a control. We found WISP1-overexpressed tumors were more radioresistant with significantly increased tumor volume and weight than control tumors when treated with 12 Gy of radiation in three fractions (255.3928 mm^3^ *vs* 154.9216 mm^3^, *P*=0.029; 0.12705 g *vs* 0.0687 g, *P*=0.0134) (Fig. 5E, 5F and 5G). Up-regulation of WISP1 expression alone had no significant promotion effect on the volume of KYSE-150 xenograft tumors (459.1966 mm^3^ *vs* 434.0424 mm^3^, *P*=0.0797; 0.4098 g *vs* 0.3862 g, *P*=0.1315) (Fig. 5E, 5F and 5G). In general, these data with three xenograft tumors together confirmed that WISP1 mediated radioresistance of ESCC in vivo as it did *in vitro*.

### WISP1 repressed γ-H2AX expression and activated PI3K kinase

**Fig.6:**
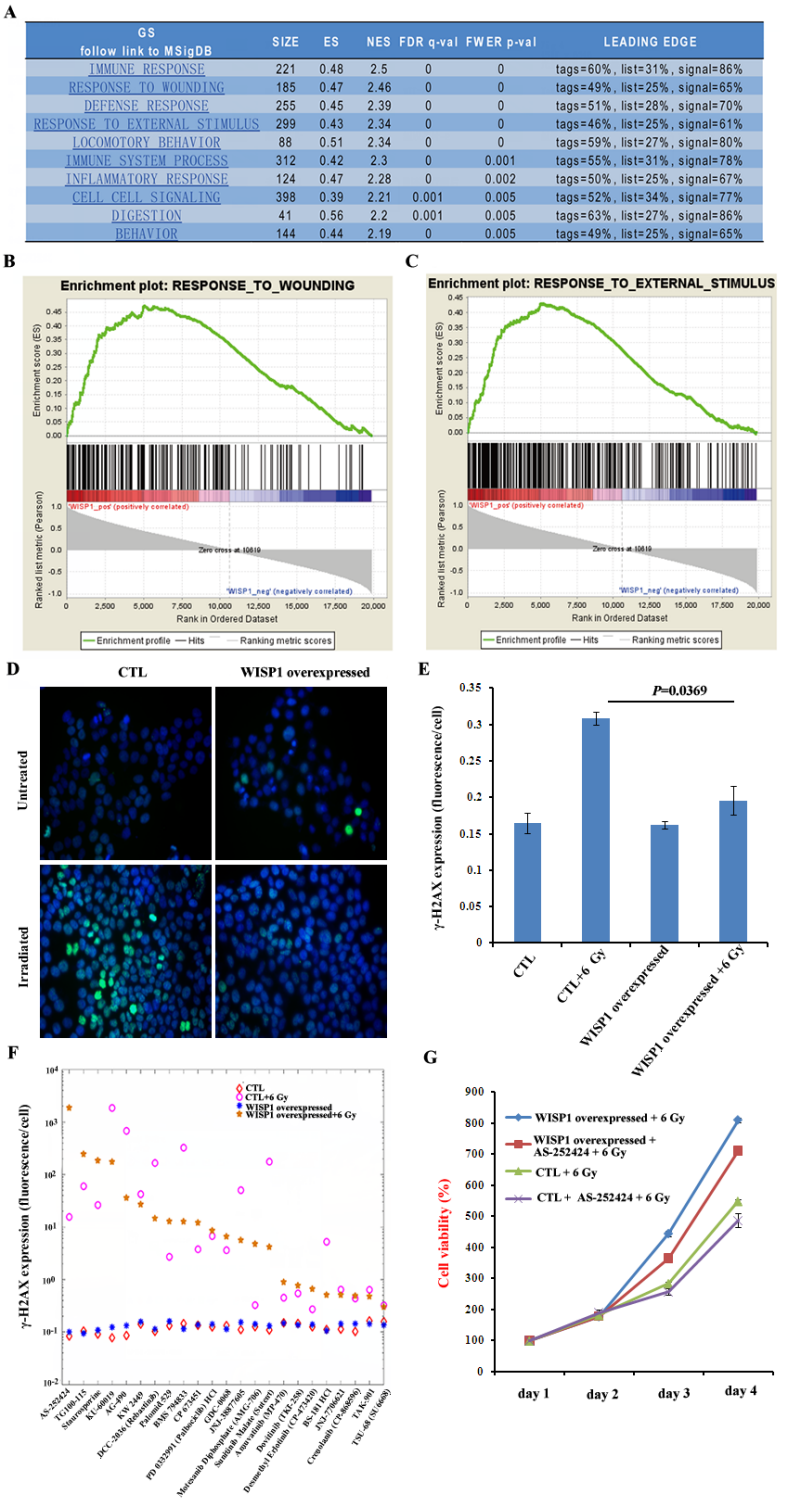
WISP1 inhibited irradiation-induced DNA damage and activated anti-apoptotic PI3K kinase A. The analysis of enriched pathways in WISP1-overexpressed esophageal cancer tissues by use of bioinformatics tool GSEA. B. The result of GSEA analysis of the relationship between WISP1 and wounding response pathway. C. The result of GSEA analysis of the relationship between WISP1 and cell response to external stimulus. D. Immunofluorescence analysis of γ-H2AX expression 30 min after 6 Gy of radiation in control KYSE-150 cells transduced with empty vector and in WISP1-overexpressed KYSE-150 cells transduced with WISP1 cDNA. *Magnification*: 40 ×. E. Quantitative analysis of γ-H2AX expression in “D”. F. Immunofluorescence analysis of γ-H2AX expression 30 min after 6 Gy of radiation in empty vector-transduced control KYSE-150 cells and in WISP1-overexpressed KYSE-150 cells with or without pretreatment with 30 μM of indicated kinase inhibitors 4 h before radiation. G. Detection of the growth of empty vector-transduced control KYSE-150 cells and WISP1-overexpressed KYSE-150 cells with or without pretreatment with 30 μM of PI3K kinase inhibitor AS-252424 4 h before 6 Gy of radiation by MTT assay.

WISP1 was demonstrated to inhibit cell apoptosis following DNA damage through inhibition of cytochrome c release and up-regulation of Bcl-xl expression [13]. In our context, to clarify the mechanisms by which WISP1 mediated radioresistance, the pathways that were enriched in WISP1-overexpressed esophageal cancer tissues were systemically analyzed by use of bioinformatics tool GSEA. We found the top four enriched pathways were about immune response, response to wound, defense response and response to external stimulus (Fig. 6A). Particularly, there were 185 genes involved in tumor response to wound, and 299 genes in tumor response to external stimulus (Fig. 6A, 6B and 6C), suggesting that WISP1 may be closely related to cell defense response to wound. In our context, we investigated whether WISP1 participated in cell response to irradiation-induced double-strand breaks (DSBs) of DNA, one known major wound for irradiated cells. We found the expression level of γ-H2AX, a marker of DSBs, in WISP1-overexpressed KYSE-150 cells was significantly lower than in control cells 30 min after 6 Gy of radiation (Fig. 6D and 6E, *P*=0.0369). This DNA damage inhibitory effect of WISP1 would greatly reduce irradiation-induced cell death. Furthermore, WISP1-regulated anti-apoptotic pathways in ESCC were also investigated by high content screening of kinases inhibitor library (Supplementary Table S5). We found treatment with 30 μM of PI3K kinase inhibitor AS-252424 4 h before radiation greatly reversed the inhibitory effect of WISP1 on irradiation-induced γ-H2AX expression in KYSE-150 cells, while AS-252424 alone had little effect on γ-H2AX expression (Fig. 6F). This result suggested that WISP1 mediated radioresistance of ESCC possibly through activation of anti-apoptotic PI3K kinase. By western blotting analysis, PI3K kinase was found to be obviously activated in WISP1-overexpressed KYSE-150 cells. Inhibition of PI3K kinase activity by treatment with 30 μM of its inhibitor AS-252424 greatly enhanced cell growth inhibitory effect of radiation on WISP1-overexpressed KYSE-150 cells (Supplementary Fig. S3A and Fig. 6G). Moreover, this combination treatment with AS-252424 and radiation obviously promoted γ-H2AX expression in WISP1-overexpressed KYSE-150 cells compared with radiation alone (Supplementary Fig. S3B). These results may together prove that WISP1 mediated radioresistance of ESCC mainly by repression of irradiation-induced DNA damage and activation of anti-apoptotic PI3K kinase.

### LncRNA BOKAS promoted WISP1 up-regulation and radioresistance

**Fig.7:**
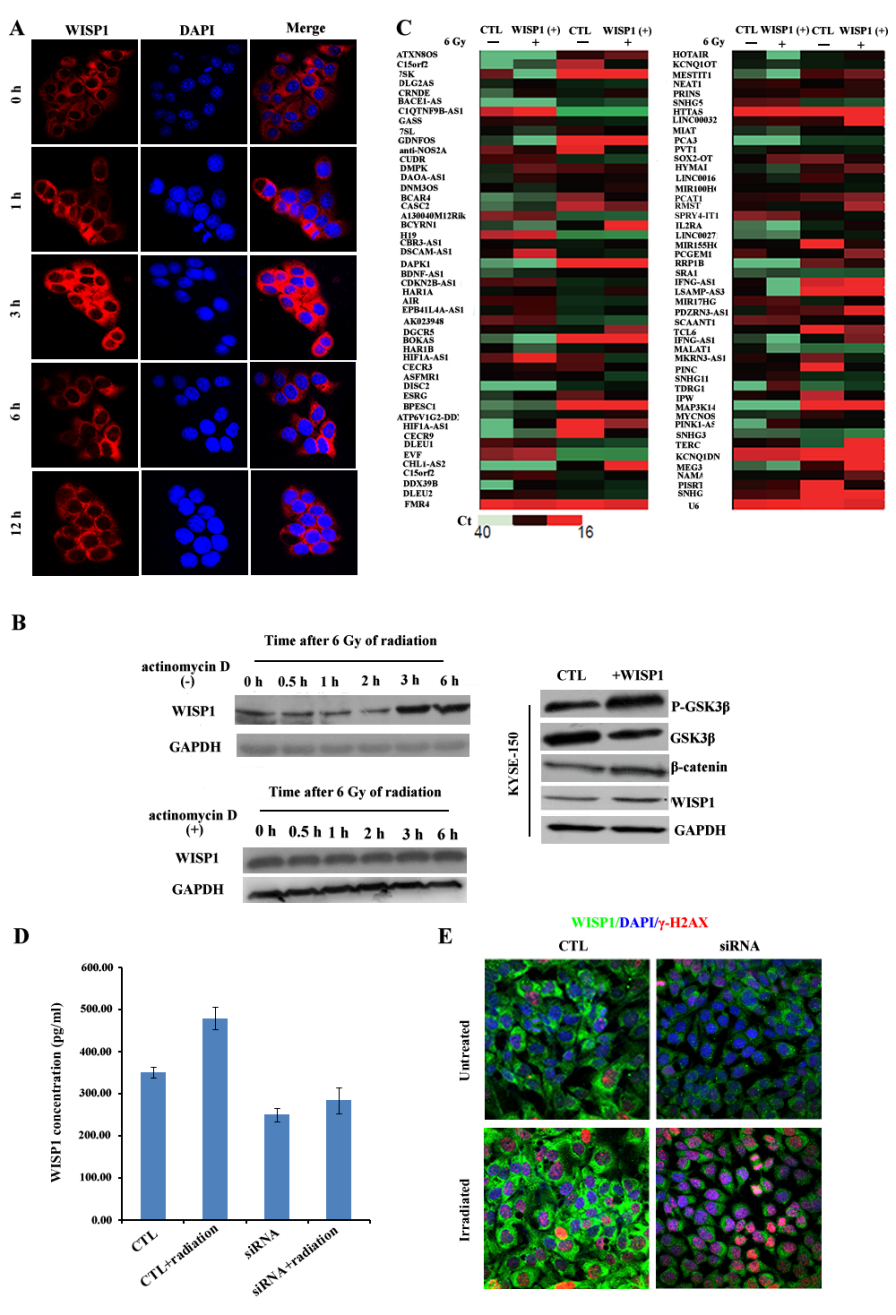
LncRNA BOKAS promoted WISP1 up-regulation and radioresistance A. The expression of WISP1 protein in KYSE-150 cells after 6 Gy of radiation by immunofluorescence analysis. B. Western blotting analysis of WISP1 expression in KYSE-150 cells treated with or without 10 μg/mL of actinomycin D 1 h before 6 Gy of radiation and the expressions of P-GSK3β, GSK3β, β-catenin and WISP1 in KYSE-150 cells treated with or without 2 μg/mL of recombinant WISP1 protein for 24 h. GAPDH was used as a loading control. C. qRT-PCR analysis of 94 cancer-related LncRNAs in control KYSE-150 cells transduced with empty vector and in WISP1-overexpressed KYSE-150 cells transduced with WISP1 cDNA before radiation and 30 min after 6 Gy of radiation. D. Detection of WISP1 concentration in control KYSE-150 cells transfected with empty vector and in KYSE-150 cells transfected with 100 nM of BOKAS-targeting siRNA before radiation and 30 min after 6 Gy of radiation by ELISA assay. E. Immunofluorescence analysis of γ-H2AX expression in control KYSE-150 cells transfected with empty vector and in KYSE-150 cells transfected with 100 nM of BOKAS-targeting siRNA before radiation and 30 min after 6 Gy of radiation.

To further clarify the mechanisms of WISP1-mediated radioresistance in ESCC, the expression pattern of WISP1 following radiation was also investigated in KYSE-150 cells. We found the level of WISP1 expression in KYSE-150 cells peaked 3 h after 6 Gy of radiation (Fig. 7A and 7B). However, when the transcription process was blocked by treatment with 10 μg/mL of actinomycin D 1 h before 6 Gy of radiation, the level of WISP1 expression in KYSE-150 cells did not increase (Supplementary Fig. S4 and Fig. 7B). Therefore, we proposed that irradiation-induced WISP1 up-regulation occurred at transcription or post-transcription level. Long non-coding (Lnc) RNAs have recently emerged as important regulators of their target genes expressions at transcription or post-transcription level [18]. In our study, the LncRNA that was associated with irradiation-induced WISP1 up-regulation was explored. We examined the expressions of 94 cancer-related LncRNAs in LncRNAs & disease database in WISP1-overexpressed KYSE-150 cells and in control cells before and 30 min after 6 Gy of radiation. There were 14 LncRNAs up-regulated and 5 LncRNAs down-regulated 30 min after 6 Gy of radiation, all of which showed more than 2-fold change in expression level (Fig. 7C). Interestingly, WISP1 was found to play an opposite effect on radiation regulation of LncRNAs expression (Fig. 7C). Among these LncRNAs, BOKAS was found to be closely related to irradiation-induced WISP1 up-regulation. When down-regulating BOKAS expression by transfection with BOKAS-targeting siRNA, the level of WISP1 expression in KYSE-150 cells decreased in a siRNA dose-dependent manner (Supplementary Fig. S5). We found the level of WISP1 expression in KYSE-150 cells decreased from 351.09 pg/ml to 99.3578 pg/ml when transfecting 100 nM of BOKAS-targeting siRNA (*P*=0.0242) (Fig. 7D). Furthermore, the level of WISP1 expression in BOKAS siRNA-transfected KYSE-150 cells was only slightly up-regulated from 99.3578 pg/ml to 116.14 pg/ml 30 min after 6 Gy of radiation (Fig. 7D, *P*=0.2791). In comparison, the level of WISP1 expression in empty vector-transfected KYSE-150 cells increased from 351.09 pg/ml to 569.93 pg/ml 30 min after 6 Gy of radiation (Fig. 7D, P=0.0136). When WISP1 up-regulation was attenuated by transfection with 100 nM of BOKAS-targeting siRNA, irradiation-induced DNA damage was greatly enhanced in KYSE-150 cells (Fig. 7E). These results indicated that BOKAS promoted WISP1 up-regulation and resultant radioresistance.

WISP1 was reported to induce its own expression in adult mouse cardiomyocytes by phosphorylation and inactivation of GSK3β, a key enzyme that inhibits Wnt/β-catenin pathway [19, 20]. In our study, we investigated whether WISP1 could also induce its own expression in KYSE-150 cells. By treatment with 2 μg/mL of recombinant WISP1 protein for 24 h, the expressions of β-catenin and WISP1 in KYSE-150 cells both increased as a result of increased phosphorylation and degradation of GSK3β (Fig. 7B). This positive feedback loop of WISP1 expression may further enhance refractory radioresistance of ESCC.

## DISCUSSION

Radiotherapy is often used for the treatment of ESCC patients. However, the prognosis is very poor [2, 3]. The mechanisms of radioresistance are known to alter in different cell types and even in different subpopulations within the tumor [21-24]. Although there have been several studies performed to explore the radiosensitizing targets, it still remained a tough problem to overcome ESCC radioresistance [25-32]. Our study revealed WISP1 as an excellent predictor of radiotherapy outcomes and also as a potential target to reverse ESCC radioresistance. Due to great difference of WISP1 expression in esophageal carcinoma tissues and in adjacent normal tissues, targeting WISP1 to reverse radioresistance would be more tumor-specific and thereby have minor adverse effect on normal tissues. Furthermore, WISP1 allocated to the CCN family that contains six members with highly conserved structure is a secreted matricellular protein, which may facilitate the development of antibody-based radiosensitizing therapies in ESCC [33]. All of these advantages of WISP1 may accelerate establishment of personalized radiotherapeutic regimens from which patients would acquire more therapeutic benefits.

Several lines of evidence have demonstrated that DNA damage response was closely associated with cell radiosensitivity [34-38]. DNA double-strand breaks (DSBs) is recognized as the main form of DNA damage induced by radiation [39]. γ-H2AX, a known marker of DSBs, is highly expressed following radiation and plays vital roles in initiating DNA damage response by interaction with the MRE11-RAD50–NBS1 (MRN) complex, which activates the ataxia telangiectasia mutated (ATM) kinase and/or ataxia telangiectasia and Rad3-related (ATR) kinase [40]. When activated, these two kinases phosphorylate their downstream genes such as BRCA1, p53, Chk1 and Chk2 to induce cell cycle arrest and/or cell apoptosis. In our study, WISP1 was found to inhibit irradiation-induced γ-H2AX expression, leading to greatly attenuated DNA damage response and reduced inhibitory effect of radiation on ESCC. Bio-informatics analysis also proved that WISP1 was closely related to cell defense response to external stimulus. Furthermore, WISP1 was found to mediate radioresistance by activation of anti-apoptotic PI3K kinase. It has been reported that PI3K kinase was associated with three known major radioresistance mechanisms including intrinsic radioresistance, tumor cell proliferation and hypoxia [4]. Inhibition of PI3K kinase activity could enhance tumor radiosensitivity [41-43]. Our study revealed that PI3K kinase was a downstream effector of WISP1 in the development of ESCC radioresistance. Although WISP1 mediated radioresistance of ESCC, it was reported to play positive roles in lung cancer by inhibition of cell motility and invasion [44]. In other human cancers such as endometrial endometrioid adenocarcinoma, breast cancer and colorectal cancer, the expression of WISP1 was also associated with poor prognosis of patients [45-47]. Thus, WISP1 regulated cancer progression in a cell type-dependent manner and may play contradictory roles among different cancers.

The expression of WISP1 during embryonic development and in adult normal tissues is exquisitely controlled by a complicated regulatory machinery. When deregulated, aberrant expression of WISP1 often led to accelerated cell growth and enhanced motility and invasion [48-50]. However, the mechanisms that caused WISP1 expression disordered have remained mostly unknown. In our study, we found WISP1 was significantly up-regulated following radiation at transcription or post-transcription level. LncRNAs are emerging regulators of their target genes expressions at transcription or post-transcription level. There were studies demonstrating that LncRNAs have several important biological functions such as cell cycle regulation, chromatin modification and nuclear-cytoplasmic tracking [51-53]. Radiation was reported to induce significant up-regulation or down-regulation of LncRNAs [54-57]. However, whether LncRNA regulated WISP1 expression and was involved in cell radioresponse has been less studied. LncRNA BOKAS was discovered as a natural antisense transcript of BOK, a member belonging to the pro-apoptotic Bcl-2 family. BOKAS was reported to be expressed in certain cancer tissues and in testis, but not other normal adult tissues [58]. Overexpression of BOKAS was demonstrated to inhibit Bok-induced apoptosis in HeLa cells. In our study, BOKAS was demonstrated to be up-regulated following radiation and promoted WISP1 expression and resultant radioresistance. However, how BOKAS promoted WISP1 expression needs to be further clarified in the future work.

Furthermore, our study demonstrated that WISP1 promoted phosphorylation and inactivation of GSK3β, a key enzyme that inhibits activation of Wnt/β-catenin pathway. Less active GSK3β enhanced activation of Wnt/β-catenin pathway and resultant WISP1 expression; in turn, increased WISP1 expression further led to phosphorylation and inactivation of GSK3β, which eventuated in a strong positive feedback loop of WISP1 expression and concomitant refractory radioresistance. Wnt/β-catenin pathway was reported to inhibit GSK3β activity through an unknown mechanism [59]. Here, our study revealed that WISP1, a downstream target gene of Wnt/β-catenin pathway, was at least in part responsible for attenuated activity of GSK3β. GSK3β is known to be a specific substrate of Akt kinase. When signaling cascade triggered activation of Akt, GSK3β would be phosphorylated and inactivated by the interaction with Akt [59]. Since WISP1 activation of Akt kinase has been reported previously [13, 19], we therefore proposed that WISP1 inhibited GSK3β activity possibly through activation of Akt. Together, our data highlighted WISP1 as a promising target to reverse ESCC radioresistance, and it deserves further exploration in preclinical studies.

## MATERIALS AND METHODS

### Cell culture and agents

The human esophageal squamous cancer cell line KYSE-150 was obtained from the Japanese Collection of Research Bioresources (JCRB, Osaka, Japan). The radioresistant esophageal cancer cell line KYSE-150R was established from KYSE-150 by multiple fractionated radiation and had been used in our previous study [15]. The other esophageal squamous cancer cell lines KYSE-30 and KYSE-180 were obtained from American Type Culture Collection. All of esophageal squamous cancer cell lines used in our study were cultured in RPMI-1640 medium (Gibco, Life Technologies Inc., Grand Island, NY, USA) supplemented with 10 % of fetal bovine serum and incubated at 37 ^o^C in 5 % CO_2_/95 % air.

Antibodies against γ-H2AX, p-PI3K (Tyr199/458) and PI3K were purchased from Cell Signaling Technology (Beverly, MA, USA). Anti-WISP1 antibody α-WISP1 and recombinant WISP1 protein were purchased from Abcam (Cambridge, MA, USA). Antibodies against phospho-GSK3β (Ser9) and total GSK3β were obtained from Epitomics (Burlingame, CA, USA). Antibody against total GAPDH was purchased from Santa Cruz Biotechnology (Dallas, TX, USA). Actinomycin D was purchased from BangYi Biotechnology Co. (Shanghai, China).

### Animals and clinical specimens of ESCC patients

6-week-old female BALB/c nude mice were purchased from Vital River (Beijing, China) and maintained under standard conditions in Experimental Animal Center in Zhejiang Chinese Medicine University. All of the animal protocols in our study were in accordance with the institutional animal welfare guidelines of Zhejiang Chinese Medicine University.

The surgically resected or biopsy specimens of ESCC tissues and serum samples used in our study were collected with the informed content of patients obtained. Each cohort of patients analyzed provided the following information: age, gender, TNM stage and therapeutic regimens received as shown in Supplemental Information. The tissue chips consisting of various independent groups of 50 primary ESCC specimens and matched non-neoplastic tissues were purchased from Alenabio Biotechnology Co (Xi'an, China).

### RNA extraction, qRT-PCR and LncRNA PCR array

Total RNA was extracted from cancer cells using the Trizol Reagent (Invitrogen life technologies, Carlsbad, CA, USA) following the manufacturer's instructions. Reverse transcription was performed with Fermentas K1622 following the manufacturer's instructions. Quantitative RT-PCR (qRT-PCR) was conducted using SYBR green (Abgene, Epsom, UK) according to the manufacturer's instructions. LncRNA PCR array (Supplementary Table S6) (Funeng biology Co, Shanghai, China) containing 94 cancer-related LncRNAs in LncRNAs & disease database were used following the manufacturer's instructions to investigate the association of LncRNAs with radiation response.

### Western blotting analysis

Protein expression was analyzed by western blotting according to the method described by Meihua Sui *et al* [16]. Briefly, cells after indicated treatments were harvested by trypsin-EDTA exposure and washed twice with ice-cold PBS before adding into protein extraction buffer. Equal amount of protein was fractionated on 12 % SDS-PAGE gel and transferred to polyvinylidence difluoride membranes. The membranes were incubated with the indicated primary and secondary antibodies. Proteins were ultimately visualized by enhanced chemiluminescence and autoradiography (ECL; Thermon Scientific, Waltham, MA, UK).

### Clonogenic survival assay

Exponentially growing esophageal squamous cancer cells were seeded into six-wells plate. After 24 h of incubation, adhesive cells receiving indicated pretreatments were exposed to radiation at 0 Gy, 2 Gy, 4 Gy, 6 Gy and 8 Gy with an average dose rate of 100 cGy/min. Then, the cells were cultured for 10 days at 37 ^o^C in a 5% CO_2_ environment to allow colony formation. Only colonies containing ≥ 50 cells were counted as clonogenic survivors. Untreated cells were chosen as a control.

### 3-(4, 5-Dimethylthiazol-2-yl)-2,5-diphenyltetrazolium bromide (MTT) assay

Cell growth was determined by MTT assay. Briefly, adherent cells (5000 cells per well) were evenly plated into 96-wells plate and incubated overnight. Then, cells were exposed to different treatments. After incubation for indicated time, the medium in each well was replaced with fresh culture medium containing 1 mg/mL of MTT. The plates were incubated for additional 3 h, allowing viable cells to reduce the yellow tetrazolium salt (MTT) into dark blue formazan crystals. Finally, DMSO was added to dissolve the formazan crystals. The absorbance was determined at 562 nm with a microplate spectrophotometer.

### Immunohistochemical staining

Immunohistochemical staining of WISP1 was performed on paraffin-embedded sections of surgically resected or biopsy specimens of ESCC tissues according to standard procedures [17]. Briefly, sections of 4 um thick were deparaffinized and rehydrated trough a series of graded alcohols. Endogenous peroxidase activity was quenched with 3 % (v/v) H_2_O_2_ for 20 minutes. Antigen retrieval was performed with 6.5 mM sodium citrate, pH 6.0, in a pressure cooker. Non-specific binding was avoided by immersing sections into 3 % bovine serum albumin (BSA) in PBS for 30 min at room temperature. Then, sections were incubated with antibody against WISP1 and HPR-conjugated secondary antibody, and the color reaction was carried out by exposure to DAB. The sections were counterstained with hematoxylin for 30 s and dehydrated in alcohol before mounting. The intensity of WISP1 expression was graded as 0, negative; 1+, weak cytoplasmic staining; 2+, strong staining in less than 30 % of tumor cells; 3+, strong staining in more than 30 % of tumor cells. 0 and 1+ were defined as WISP1-negative; 2+ and 3+ as WISP1-positive. The slides were scored by a pathologist and two experienced researchers independently.

### Enzyme-linked Immunosorbent Assay (ELISA)

The concentration of WISP1 in serum of ESCC patients was determined by sandwich ELISA kit (R&D Systems, Minneapolis, Minnesota, USA) according to the manufacturer's instructions.

### Viral transduction of WISP1 shRNA or cDNA

To establish WISP1-silenced or overexpressed cells, KYSE-150 cells were transduced with lentivirus vectors containing WISP1-targeting shRNA (5′-GCTGTGAGTGCTGTAAGATGT-3′) or WISP1 cDNA. Cells transduced with empty vector were used as negative control. WISP1-silenced or overexpressed cells were those clones that survived after treatment with 1 μg/mL of puromycin and further confirmed by western blotting.

### Transfection of BOKAS-targeting siRNA into KYSE-150 cells

KYSE-150 cells were seeded into 6-wells plate (1×10^5^ cells/well) and cultured for 24 h. Adherent cells were incubated with the complex of BOKAS-targeting siRNA and lipofectamin at a final siRNA concentration of 1 nM, 10 nM or 100 nM. 6 h later, the transfection medium was replaced with fresh RPMI-1640 medium and cells were cultured for additional 48 h. Cells transfected with empty vector were used as negative control. The mRNA level of BOKAS in KYSE-150 cells after transfection with siRNA or empty vector was detected by qRT-PCR analysis.

### Immunofluorescence detection of γ-H2AX expression

Cells were seeded into 96-wells plate and exposed to 6 Gy of radiation after adherent growth. 30 minutes later, cells were fixed with acetone/methanol (1:1), and permeabilized with 0.1 % Triton-X100 in PBS. Non-specific binding was blocked with 3 % (m/v) BSA in PBS. Then, the cells were incubated with antibody against γ-H2AX for 2 h in PBS containing 0.1 % (m/v) BSA. Indirect immunofluorescence was performed by incubation with Alexa Fluor 488-conjugated secondary antibodies (Zymed; Invitrogen). Cell nucleus was stained with 1 μg/mL of DAPI. Immunofluorescence images were taken using a confocal laser scanning microscope. Data was shown as average intensity of γ-H2AX fluorescence per cell.

### High content screening of kinases inhibitors library

Kinases inhibitors library shown in Supplementary Table S5 was purchased from Selleck (Houston, TX, USA). The compounds were dissolved in DMSO to make 30 mM of stock solution and kept in −20 °C until use. The tested cells were seeded into 384-wells plate (5×10^3^ per well) and cultured for adherent growth. After 4 h of incubation with 30 μM of various kinases inhibitors, cells were treated with 6 Gy of radiation, and detection of γ-H2AX expression was performed 30 min later as described above.

### Xenograft transplantation and therapy

To develop xenograft tumors, *in vitro* growing esophageal squamous cancer cells were harvested by exposure to trypsin-EDTA, washed with ice-cold PBS and implanted into the right flanks of female BALB/c nude mice (1.0×10^5^ cells in 100 μl PBS). When xenograft tumors had reached a mean diameter of around 0.5 cm, mice were randomly assigned into different groups (five mice in each group). Tumors were treated with 12 Gy of radiation in three fractions every four days, 4 μg/mL of anti-WISP1 antibody or 2 μg/mL of recombinant WISP1 protein daily for 12 consecutive days, alone or in their combinations. Each animal was earmarked and followed individually throughout the experiments. Tumor volume (mm^3^) was calculated using the following formula: V(mm^3^)= A(mm)×B(mm)^2^/2, where A and B were the longest and widest diameter of tumor, respectively, and measured every two days by a caliper.

### Statistics analysis

All of the experiments in our study were independently performed in triplicate and data were presented as means ± SD. Statistical analyses were performed with SPSS software 16.0 (SPSS). Univariate survival analyses were performed with Kaplan–Meier method and log-rank tests. The other statistical analyses were performed with Student's t-test. Differences were considered statistically significant at a level of p<0.05.

## SUPPLEMENTARY FIGURES



## References

[1] Enzinger PC, Mayer RJ (2003). Medical progress - Esophageal cancer. New ENJL J MED.

[2] Cooper JS, Guo MD, Herskovic A, Macdonald JS, Martenson JA, Al-Sarraf M, Byhardt R, Russell AH, Beitler JJ, Spencer S, Asbell SO, Graham MV, Leichman LL (1999). Chemoradiotherapy of locally advanced esophageal cancer - Long-term follow-up of a prospective randomized trial (RTOG 85-01). Jama-J Am Med Assoc.

[3] Minsky BD, Pajak TF, Ginsberg RJ, Pisansky TM, Martenson J, Komaki R, Okawara G, Rosenthal SA, Kelsen DP (2002). INT 0123 (radiation therapy oncology group 94-05) phase III trial of combined-modality therapy for esophageal cancer: High-dose versus standard-dose radiation therapy. J Clin Oncol.

[4] Bussink J, van der Kogel AJ, Kaanders J (2008). Activation of the PI3-K/AKT pathway and implications for radioresistance mechanisms in head and neck cancer. Lancet Oncol.

[5] De Bacco F, Luraghi P, Medico E, Reato G, Girolami F, Perera T, Gabriele P, Comoglio PM, Boccaccio C (2011). Induction of MET by Ionizing Radiation and Its Role in Radioresistance and Invasive Growth of Cancer. J Natl Cancer I.

[6] Uzawa K, Ishigami T, Fushimi K, Kawata T, Shinozuka K, Kasamatsu A, Sakamoto Y, Ogawara K, Shiiba M, Bukawa H, Ito H, Tanzawa H (2011). Targeting fibroblast growth factor receptor 3 enhances radiosensitivity in human squamous cancer cells. Oncogene.

[7] Kim Y, Kim KH, Lee J, Lee YA, Kim M, Lee SJ, Park K, Yang H, Jin J, Joo KM, Nam DH (2012). Wnt activation is implicated in glioblastoma radioresistance. Lab Invest.

[8] Chen MS, Woodward WA, Behbod F, Peddibhotla S, Alfaro MP, Buchholz TA, Rosen JM (2007). Wnt/beta-catenin mediates radiation resistance of Sca1(+) progenitors in an immortalized mammary gland cell line. J Cell Sci.

[9] Chang HW, Roh JL, Jeong EJ, Lee SW, Kim SW, Choi SH, Park SK, Kim SY (2008). Wnt signaling controls radiosensitivity via cyclooxygenase-2-mediated Ku expression in head and neck cancer. Int J Cancer.

[10] Pennica D, Swanson TA, Welsh JW, Roy MA, Lawrence DA, Lee J, Brush J, Taneyhill LA, Deuel B, Lew M, Watanabe C, Cohen RL, Melhem MF, Finley GG, Quirke P, Goddard AD (1998). WISP genes are members of the connective tissue growth factor family that are up-regulated in wnt-1-transformed cells and aberrantly expressed in human colon tumors. P Natl Acad Sci USA.

[11] Berschneider B, Konigshoff M (2011). WNT1 inducible signaling pathway protein 1 (WISP1): A novel mediator linking development and disease. Int J Biochem Cell B.

[12] Xu LF, Corcoran RB, Welsh JW, Pennica D, Levine AJ (2000). WISP-1 is a Wnt-1-and beta-catenin-responsive oncogene. Genes Dev.

[13] Su F, Overholtzer M, Besser D, Levine AJ (2002). WISP-1 attenuates p53-mediated apoptosis in response to DNA damage trough activation of the Akt kinase. Genes Dev.

[14] Nagai Y, Watanabe M, Ishikawa S, Karashima R, Kurashige J, Iwagami S, Iwatsuki M, Baba Y, Imamura Y, Hayashi N, Baba H (2011). Clinical Significance of Wnt-induced Secreted Protein-1 (WISP-1/CCN4) in Esophageal Squamous Cell Carcinoma. Anticancer Res.

[15] Jing Z, Gong L, Xie CY, Zhang L, Su HF, Deng X, Wu SX (2009). Reverse resistance to radiation in KYSE-150R esophageal carcinoma cell after epidermal growth factor receptor signal pathway inhibition by cetuximab. Radiother Oncol.

[16] Sui M, Huang Y, Park BH, Davidson NE, Fan W (2007). Estrogen receptor alpha mediates breast cancer cell resistance to paclitaxel through inhibition of apoptotic cell death. Cancer Res.

[17] Konigshoff M, Kramer M, Balsara N, Wilhelm J, Amarie OV, Jahn A, Rose F, Fink L, Seeger W, Schaefer L, Gunther A, Eickelberg O (2009). WNT1-inducible signaling protein-1 mediates pulmonary fibrosis in mice and is upregulated in humans with idiopathic pulmonary fibrosis. J Clin Invest.

[18] Nishimoto Y, Nakagawa S, Hirose T, Okano HJ, Takao M, Shibata S, Suyama S, Kuwako K, Imai T, Murayama S, Suzuki N, Okano H (2013). The long non-coding RNA nuclear-enriched abundant transcript 1_2 induces paraspeckle formation in the motor neuron during the early phase of amyotrophic lateral sclerosis. Mol Brain.

[19] Venkatesan B, Prabhu SD, Venkatachalam K, Mummidi S, Valente AJ, Clark RA, Delafontaine P, Chandrasekar B (2010). WNT1-inducible signaling pathway protein-1 activates diverse cell survival pathways and blocks doxorubicin-induced cardiomyocyte death. Cell Signal.

[20] Clevers H (2006). Wnt/beta-catenin signaling in development and disease. Cell.

[21] Ke C, Tran K, Chen Y, Di Donato AT, Yu L, Hu Y, Linskey ME, Wang PH, Limoli CL, Zhou YH (2014). Linking differential radiation responses to glioma heterogeneity. Oncotarget.

[22] Lin HY, Hung SK, Lee MS, Chiou WY, Huang TT, Tseng CE, Shih LY, Lin RI, Lin JM, Lai YH, Chang CB, Hsu FC, Chen LC, Tsai SJ, Su YC, Li SC (2014). DNA methylome analysis identifies epigenetic silencing of FHIT as a determining factor for radiosensitivity in oral cancer: an outcome-predicting and treatment-implicating study. Oncotarget.

[23] Lv P, Wang Y, Ma J, Wang Z, Li JL, Hong CS, Zhuang Z, Zeng YX (2014). Inhibition of protein phosphatase 2A with a small molecule LB100 radiosensitizes nasopharyngeal carcinoma xenografts by inducing mitotic catastrophe and blocking DNA damage repair. Oncotarget.

[24] Meng J, Li P, Zhang Q, Yang Z, Fu S (2014). A radiosensitivity gene signature in predicting glioma prognostic via EMT pathway. Oncotarget.

[25] Qian D, Zhang B, Zeng XL, Le Blanc JM, Guo YH, Xue C, Jiang C, Wang HH, Zhao TS, Meng MB, Zhao LJ, Hao JH, Wang P, Xie D, Lu B, Yuan ZY (2014). Inhibition of human positive cofactor 4 radiosensitizes human esophageal squmaous cell carcinoma cells by suppressing XLF-mediated nonhomologous end joining. Cell Death Dis.

[26] Zhang C, Yang X, Zhang Q, Guo Q, He J, Qin Q, Zhu HC, Liu J, Zhan LL, Lu J, Liu ZM, Xu LP, Ma JX, Dai SB, Cheng HY, Sun XC (2014). STAT3 inhibitor NSC74859 radiosensitizes esophageal cancer via the downregulation of HIF-1 alpha. Tumor Biol.

[27] Zhu HG, Yang X, Liu J, Ge YY, Qin Q, Lu J, Zhan LL, Liu ZM, Zhang H, Chen XC, Zhang C, Xu LP, Cheng HY, Sun XC (2014). Melittin radiosensitizes esophageal squamous cell carcinoma with induction of apoptosis *in vitro* and *in vivo*. Tumor Biol.

[28] Qin Q, Cheng HY, Lu J, Zhan LL, Zheng JC, Cai J, Yang X, Xu LP, Zhu HC, Zhang C, Liu J, Ma JX, Zhang XZ, Dai SB, Sun XC (2014). Small-molecule survivin inhibitor YM155 enhances radiosensitization in esophageal squamous cell carcinoma by the abrogation of G(2) checkpoint and suppression of homologous recombination repair. J Hematol Oncol.

[29] Qin Q, Zuo Y, Yang X, Lu J, Zhan LL, Xu LP, Zhang C, Zhu HC, Liu J, Liu ZM, Tao GZ, Dai SB, Zhang XZ, Ma JX, Cai J, Sun XC (2014). Smac mimetic compound LCL161 sensitizes esophageal carcinoma cells to radiotherapy by inhibiting the expression of inhibitor of apoptosis protein. Tumor Biol.

[30] Luo JD, Zhou XF, Ge X, Liu PF, Cao JP, Lu XJ, Ling Y, Zhang SY (2013). Upregulation of Ying Yang 1 (YY1) suppresses esophageal squamous cell carcinoma development through heme oxygenase-1. Cancer Sci.

[31] Yang CR, Wang YD, Zhang FL, Sun GG, Li CL, Jing SW, Liu Q, Cheng YJ (2013). Inhibiting UHRF1 expression enhances radiosensitivity in human esophageal squamous cell carcinoma. Mol Biol Rep.

[32] Wang GY, Liu LY, Sharma S, Liu H, Yang WF, Sun XN, Dong QH (2012). Bmi-1 confers adaptive radioresistance to KYSE-150R esophageal carcinoma cells. Biochem Bioph Res Co.

[33] Jun JI, Lau LF (2011). Taking aim at the extracellular matrix: CCN proteins as emerging therapeutic targets. Nat Rev Drug Discov.

[34] Lundholm L, Haag P, Zong D, Juntti T, Mork B, Lewensohn R, Viktorsson K (2013). Resistance to DNA-damaging treatment in non-small cell lung cancer tumor-initiating cells involves reduced DNA-PK/ATM activation and diminished cell cycle arrest. Cell Death Dis.

[35] Sugrue T, Brown JAL, Lowndes NF, Ceredig R (2013). Multiple Facets of the DNA Damage Response Contribute to the Radioresistance of Mouse Mesenchymal Stromal Cell Lines. Stem Cells.

[36] Nagelkerke A, Bussink J, van der Kogel AJ, Sweep F, Span PN (2013). The PERK/ATF4/LAMP3-arm of the unfolded protein response affects radioresistance by interfering with the DNA damage response. Radiother Oncol.

[37] Carruthers R, Ahmed S, Chalmers AJ (2014). Inhibition of DNA damage response abrogates glioblastoma cancer stem cell radioresistance. Neuro-Oncol.

[38] Zhang P, Wei Y, Wang L, Debeb BG, Yuan Y, Zhang JS, Yuan JS, Wang M, Chen DH, Sun YT, Woodward WA, Liu YQ, Dean DC, Liang H, Hu Y, Ang KK (2014). ATM-mediated stabilization of ZEB1 promotes DNA damage response and radioresistance through CH K1. Nat Cell Biol.

[39] Santivasi WL, Xia F (2014). Ionizing Radiation-Induced DNA Damage, Response, and Repair. Antioxid Redox Sign.

[40] Hosoya N, Miyagawa K (2014). Targeting DNA damage response in cancer therapy. Cancer Sci.

[41] Schutze C, Krause M (2006). Radioresistance of K-ras-mutated human tumor cells is mediated through EGFR-dependent activation of PI3K-AKT pathway. Strahlenther Onkol.

[42] Chang L, Graham PH, Hao JL, Jie N, Bucci J, Cozzi PJ, Kearsley JH, Li Y (2014). Preclinical studies of the combination of dual PI3K/Akt/mTOR inhibitors with radiotherapy to overcome radioresistant prostate cancer. Int J Mol Med.

[43] Chang L, Graham PH, Hao J, Ni J, Bucci J, Cozzi PJ, Kearsley JH, Li Y (2014). PI3K/Akt/mTOR pathway inhibitors enhance radiosensitivity in radioresistant prostate cancer cells through inducing apoptosis, reducing autophagy, suppressing NHEJ and HR repair pathways. Cell Death Dis.

[44] Soon LL, Yie TA, Shvarts A, Levine AJ, Su F, Tchou-Wong KM (2003). Overexpression of WISP-1 down-regulated motility and invasion of lung cancer cells through inhibition of Rac activation. J Biol Chem.

[45] Tang QL, Jiang XF, Li HG, Lin ZQ, Zhou XD, Luo X, Liu L, Chen GB (2011). Expression and prognostic value of WISP-1 in patients with endometrial endometrioid adenocarcinoma. J Obstet Gynaecol Res.

[46] Davies SR, Watkins G, Mansel RE, Jiang WG (2007). Differential expression and prognostic implications of the CCN family members WISP-1, WISP-2, and WISP-3 in human breast cancer. Ann Surg Oncol.

[47] Davies SR, Davies ML, Sanders A, Parr C, Torkington J, Jiang WG (2010). Differential expression of the CCN family member WISP-1, WISP-2 and WISP-3 in human colorectal cancer and the prognostic implications. Int J Oncol.

[48] Ono M, Inkson CA, Sonn R, Kilts TM, de Castro LF, Maeda A, Fisher LW, Robey PG, Berendsen AD, Li L, McCartney-Francis N, Brown AC, Crawford NPS, Molinolo A, Jain A, Fedarko NS (2013). WISP1/CCN4: A Potential Target for Inhibiting Prostate Cancer Growth and Spread to Bone. Plos One.

[49] Chuang JY, Chang AC, Chiang IP, Tsai MH, Tang CH (2013). Apoptosis Signal-Regulating Kinase 1 Is Involved in WISP-1-Promoted Cell Motility in Human Oral Squamous Cell Carcinoma Cells. Plos One.

[50] Chang AC (2013). WISP-1 increases cell migration and ICAM-1 expression in human oral squamous cell carcinomas. Cancer Res.

[51] Wilusz JE, Sunwoo H, Spector DL (2009). Long noncoding RNAs: functional surprises from the RNA world. Gene Dev.

[52] Kim ED, Sung S (2012). Long noncoding RNA: unveiling hidden layer of gene regulatory networks. Trends Plant Sci.

[53] Yoon JH, Abdelmohsen K, Gorospe M (2013). Posttranscriptional Gene Regulation by Long Noncoding RNA. J Mol Biol.

[54] Ozgur E, Mert U, Isin M, Okutan M, Dalay N, Gezer U (2013). Differential expression of long non-coding RNAs during genotoxic stress-induced apoptosis in HeLa and MCF-7 cells. Clin Exp Med.

[55] Chaudhry MA (2013). Expression Pattern of Small Nucleolar RNA Host Genes and Long Non-Coding RNA in X-rays-Treated Lymphoblastoid Cells. Int J Mol Sci.

[56] Wang QR, Fan HN, Liu Y, Yin ZX, Cai HB, Liu J, Wang ZY, Shao M, Sun XG, Diao JX, Liu YL, Tong L, Fan Q (2014). Curcumin enhances the radiosensitivity in nasopharyngeal carcinoma cells involving the reversal of differentially expressed long non-coding RNAs. Int J Oncol.

[57] Chaudhry MA (2014). Small Nucleolar RNA Host Genes and Long Non-Coding RNA Responses in Directly Irradiated and Bystander Cells. Cancer Biothe Radio.

[58] Zhang H, Gao SP, De Geyter C (2009). A natural antisense transcript, BOKAS, regulates the pro-apoptotic activity of human Bok. Int J Oncol.

[59] Mizushima T, Nakagawa H, Kamberov YG, Wilder EL, Klein PS, Rustgi AK (2002). Wnt-1 but not epidermal growth factor induces beta-catenin/T-cell factor-dependent transcription in esophageal cancer cells. Cancer Res.

