# Supplementary material for: Targeting WISP1 to sensitize esophageal squamous cell carcinoma to irradiation
Source: Oncotarget. 2015 Jan 31;6(8):6218–34. doi: 10.18632/oncotarget.3358 (PMC4467433; doi:10.18632/oncotarget.3358)
Supplement: Supplementary file 1 [file oncotarget-06-6218-s001.pdf]

# Targeting WISP1 to sensitize esophageal squamous cell carcinoma to irradiation

## Supplementary Material

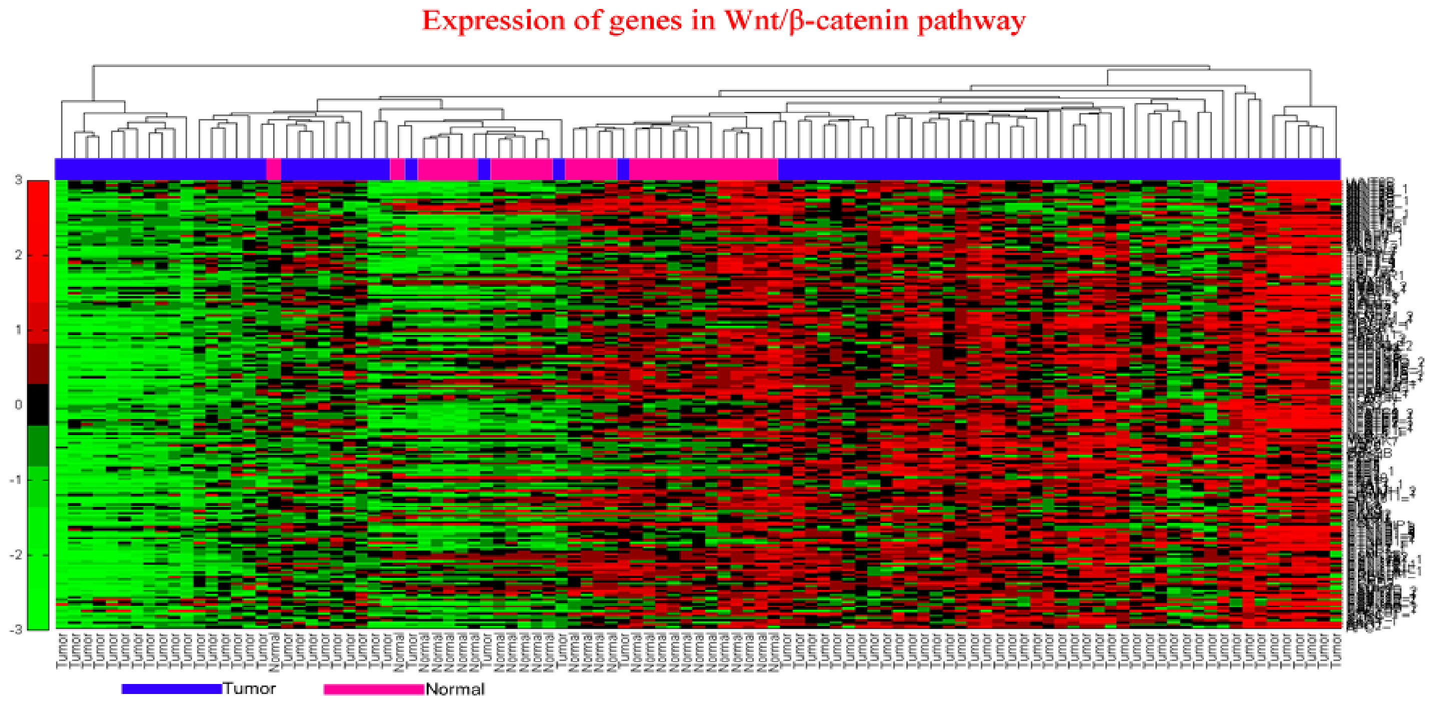


## Supplemental Figure1:

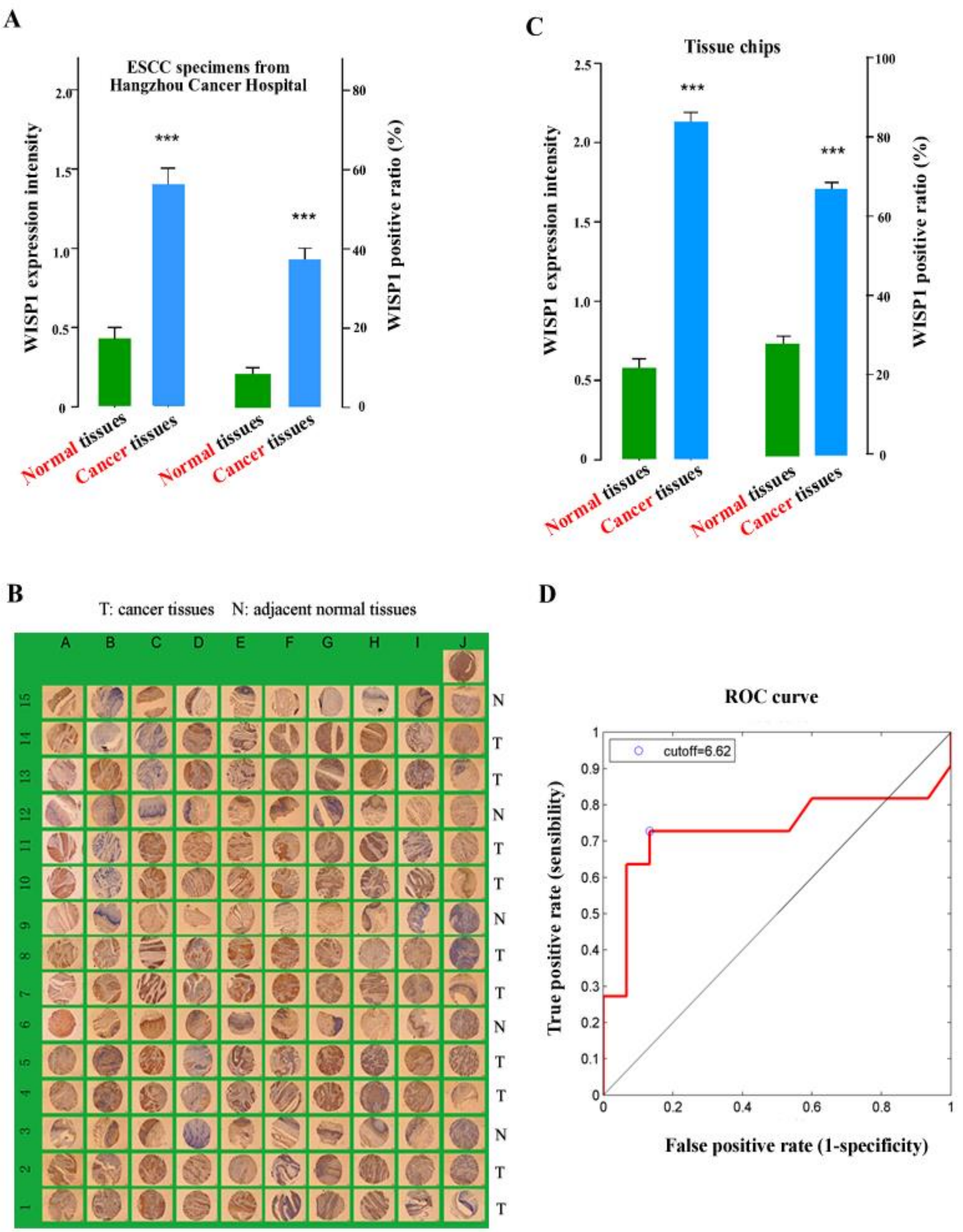


**Supplemental Figure2:**

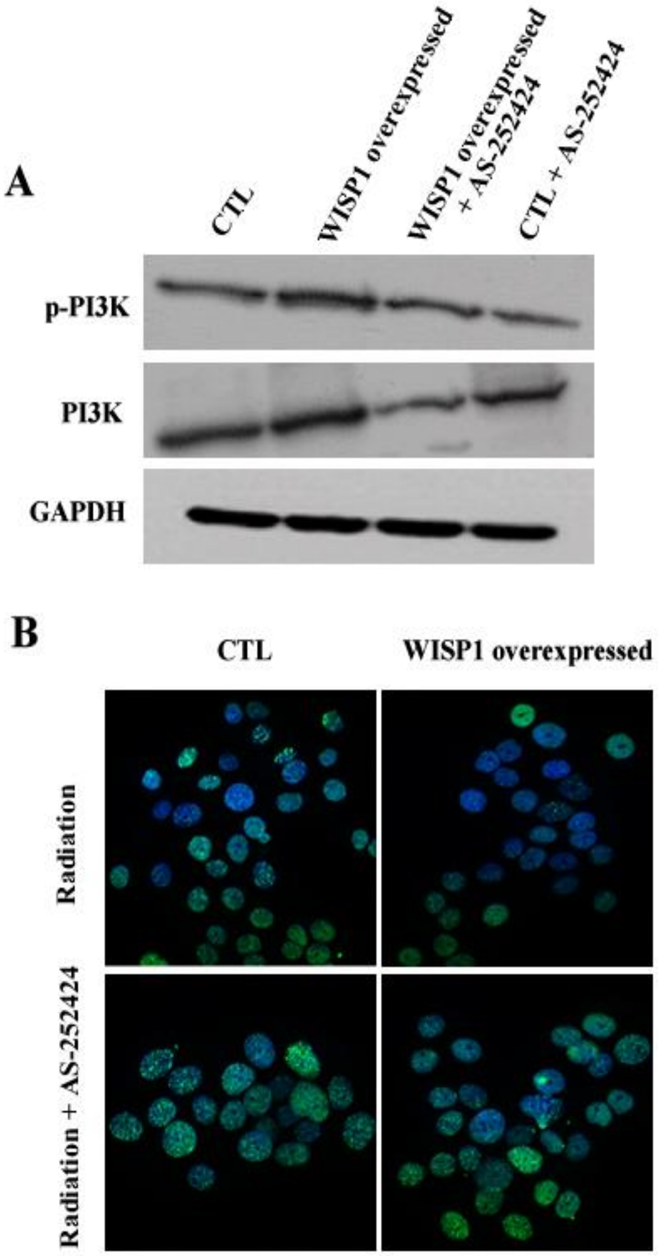


**Supplemental Figure3:**

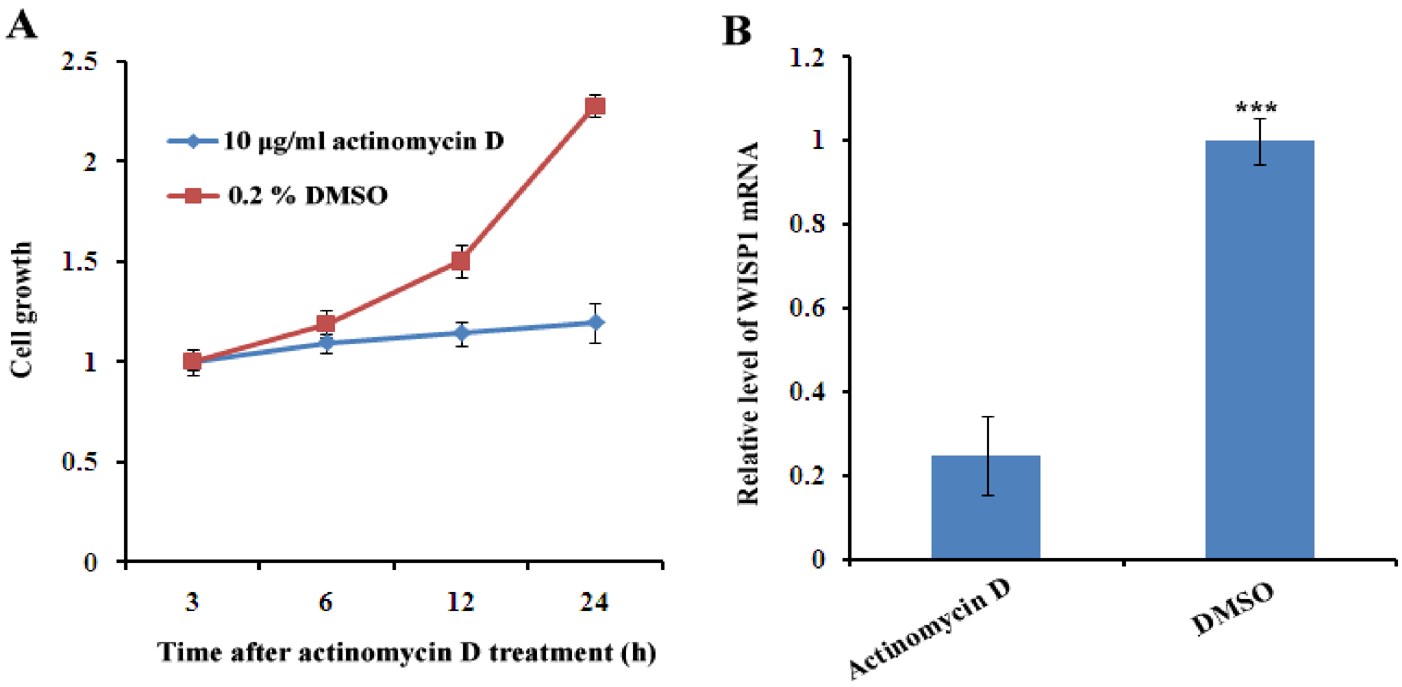


**Supplemental Figure4:**

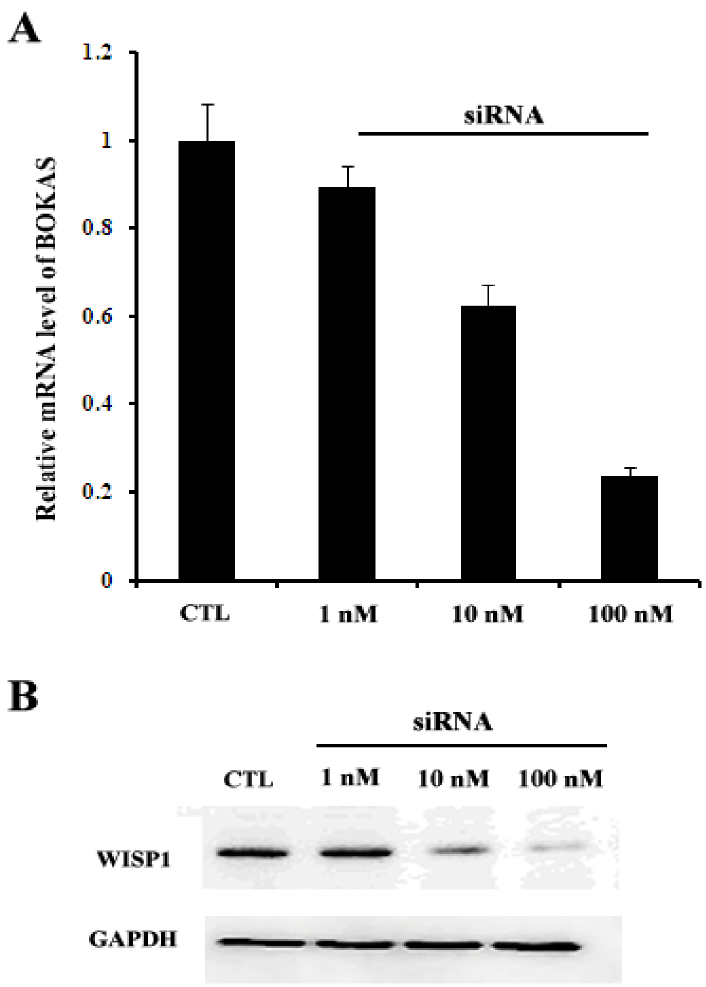


**Supplemental Figure5:**
